# Effects of Spaceflight Stressors on Brain Volume, Microstructure, and Intracranial Fluid Distribution

**DOI:** 10.1093/texcom/tgab022

**Published:** 2021-03-30

**Authors:** Jessica K Lee, Vincent Koppelmans, Ofer Pasternak, Nichole E Beltran, Igor S Kofman, Yiri E De Dios, Edwin R Mulder, Ajitkumar P Mulavara, Jacob J Bloomberg, Rachael D Seidler

**Affiliations:** Department of Applied Physiology and Kinesiology, College of Health and Human Performance, University of Florida, Gainesville, FL 32611, USA; Institute of Aerospace Medicine, German Aerospace Center, Cologne, Germany; Department of Psychiatry, University of Utah, Salt Lake City, UT 84108, USA; Deparments of Psychiatry and Radiology, Brigham and Women’s Hospital, Harvard Medical School, Boston, MA 02115, USA; KBR, Houston, TX 77002, USA; KBR, Houston, TX 77002, USA; KBR, Houston, TX 77002, USA; Institute of Aerospace Medicine, German Aerospace Center, Cologne, Germany; KBR, Houston, TX 77002, USA; NASA Johnson Space Center, Houston, TX 77058, USA; Department of Applied Physiology and Kinesiology, College of Health and Human Performance, University of Florida, Gainesville, FL 32611, USA; Norman Fixel Institute for Neurological Diseases, University of Florida, Gainesville, FL 32608, USA

**Keywords:** bed rest, brain structure, CO_2_, cognition, sensorimotor, spaceflight

## Abstract

Astronauts are exposed to elevated CO_2_ levels onboard the International Space Station. Here, we investigated structural brain changes in 11 participants following 30-days of head-down tilt bed rest (HDBR) combined with 0.5% ambient CO_2_ (HDBR + CO_2_) as a spaceflight analog. We contrasted brain changes observed in the HDBR + CO_2_ group with those of a previous HDBR sample not exposed to elevated CO_2_. Both groups exhibited a global upward shift of the brain and concomitant intracranial free water (FW) redistribution. Greater gray matter changes were seen in the HDBR + CO_2_ group in some regions. The HDBR + CO_2_ group showed significantly greater FW decrements in the posterior cerebellum and the cerebrum than the HDBR group. In comparison to the HDBR group, the HDBR + CO_2_ group exhibited greater diffusivity increases. In half of the participants, the HDBR + CO_2_ intervention resulted in signs of Spaceflight Associated Neuro-ocular Syndrome (SANS), a constellation of ocular structural and functional changes seen in astronauts. We therefore conducted an exploratory comparison compared between subjects that did and did not develop SANS and found asymmetric lateral ventricle enlargement in the SANS group. These results enhance our understanding of the underlying mechanisms of spaceflight-induced brain changes, which is critical for promoting astronaut health and performance.

## Introduction

There is accumulating evidence that spaceflight has an impact on human brain structure. We and others have shown that exposure to microgravity results in an upward displacement of the brain within the cranium (Koppelmans et al. 2016; Roberts et al. 2017), intracranial fluid redistribution (Lee, Koppelmans, et al. 2019b), and expanded ventricles (Roberts et al. 2017; Van Ombergen et al. 2019; Hupfeld, McGregor, et al. 2020). Other findings include increased somatosensory cortex gray matter (GM) volume (Koppelmans et al. 2016) and white matter changes (Alperin et al. 2017; Lee, Koppelmans, et al. 2019b).

Identifying the underlying mechanisms of these brain changes is a critical step toward understanding whether they are induced by neuroplasticity, gravitational mechanical changes, or both. In addition to microgravity, crewmembers residing in the confined compartment of the International Space Station (ISS) are exposed to elevated ambient CO_2_ levels averaging 0.5%, a level that is more than 10 times greater than terrestrial levels (0.04%) (Law et al. 2014). However, the potential interactive effect of microgravity and prolonged elevated CO_2_ on the brain is yet to be determined.

Long-duration, 6° head-down tilt bed rest (HDBR) has been widely used to simulate the physiological impacts of microgravity such as arterial pressure changes, unloading of the lower body, and cephalad fluid shifts (Hargens and Vico 2016) on Earth. We have previously shown that HDBR results in GM changes and intracranial fluid shifts similar to that seen after spaceflight (Koppelmans et al. 2017c). In the current study, we investigated the effects of an HDBR intervention combined with mild hypercapnia to closely match the conditions aboard the ISS.

We examined GM, ventricular volume, brain extracellular volume (free water), and white matter microstructural changes occurring in 11 subjects who underwent 30 days of HDBR in a controlled environment with an atmospheric CO_2_ level of 0.5% (HDBR + CO_2_). We evaluated brain changes occurring with this intervention and their associations with performance changes, focusing on measures in which we have seen significant pre-to-post bed rest changes in the same cohort (Lee et al. 2019a). In addition, we contrasted the degree of brain changes observed in the HDBR + CO_2_ group with that of our previous sample who completed 70 days of HDBR in normal atmospheric conditions (Koppelmans et al. 2017c). This is an exploratory comparison given the difference in age, sex distribution of the participants, bed rest duration, and scanning parameters between the two study protocols.

For the first time in a ground-based spaceflight analogue model, 5 of the 11 participants developed bilateral optic disc edema during the HDBR + CO_2_ intervention (Laurie et al. 2019). Optic disc edema is one of the clinical findings included in the spectrum of optic nerve and/or ocular changes seen in nearly one third of astronauts who complete long-duration missions (Lee et al. 2016), a condition coined Spaceflight Associated Neuro-ocular Syndrome (SANS) (Mader et al. 2011). Albeit preliminary due to the small sample size, this provided us with a unique opportunity to characterize the SANS subgroup differences in brain structure as a post hoc aim.

## Methods

### Participants and Study Design

#### HDBR + CO_2_

Eleven participants (6 males and 5 females) aged between 25.3 and 50.3 years at the time of admission completed the study. An additional female was enrolled but withdrew from the study on the first day of the intervention. The experiment was part of the VaPER (Vision Impairment and Intracranial Pressure and Psychological:envihab Research) bed rest campaign funded by NASA. The campaign comprises three experimental phases: a 14-day baseline data collection (BDC) phase, 30 days of strict 6° HDT with elevated CO_2_, and a 14-day recovery (R) phase. The participants resided at: envihab (the German Aerospace Center’s medical research facility in Cologne, Germany) for 58 days participating in various experiments included within the VaPER campaign. Closely replicating the average CO_2_ concentration on board the ISS (Law et al. 2014) and following the German occupational CO_2_ exposure limit, the level of atmospheric CO_2_ was set to 0.5% (3.8-mmHg partial pressure of CO_2_). The 0.5% enriched CO_2_ was continuously supplied and regulated through the ventilation system. The pre- and post-HDBR + CO_2_ data collections were conducted in ambient air (~0.04% CO_2_). As part of NASA’s standard measures assessments, blood draws were acquired 3 days prior to bed rest and on the first day after bed rest to calculate arterial partial pressure of carbon dioxide (P_a_CO_2_).

#### HDBR

Fifteen individuals who participated in a previous HDBR study conducted at the University of Texas Medical Branch, Galveston, TX, USA, were included in an exploratory comparison with the HDBR + CO_2_ subjects. The methods of this HDBR study were previously published (Yuan et al. 2016; Koppelmans et al. 2017c; Yuan, Koppelmans, Reuter-Lorenz, De Dios, Gadd, Riascos, et al. 2018a; Yuan, Koppelmans, Reuter-Lorenz, De Dios, Gadd, Wood, et al. 2018b). Participants underwent 70 days of HDBR without elevated CO_2_. The participants were all males by study design and were aged 25.7–38.5 years at the time of admission. These subjects were randomly assigned either to a no-exercise (*n* = 3), an aerobic and resistance exercise (*n* = 4), or a flywheel exercise (*n* = 8) countermeasure group. Given the similar intensity of the two exercise protocols, these groups were pooled for statistical analyses (Ploutz-Snyder et al. 2014; Koppelmans et al. 2018). The HDBR + CO_2_ subjects’ experience was most similar to the no-exercise group, with regular physiotherapy but no exercise.

All HDBR + CO_2_ and HDBR participants passed an Air Force Class III equivalent physical examination and psychological screening prior to enrollment. Both studies were part of larger bed rest projects; thus, the timelines for HDBR + CO_2_ and HDBR were determined by NASA and not matched between the two studies. The current experimental protocol was approved by the ethics commission of the local medical association (Ärztekammer Nordrhein) and institutional review boards at NASA, The University of Florida (HDBR + CO_2_), and The University of Michigan (HDBR). All subjects provided written informed consent and received monetary compensation for their participation.

The demographic information of the groups as well as the subgroups is provided in Supplementary Table 1. There were no significant differences in age, height, weight, and BMI between HDBR and HDBR + CO_2_ groups nor between the subgroups within each campaign.

#### Assessment Timeline

##### HDBR + CO_2_

Assessments were made at six times during the study: 1) two baseline measurements at 13 and 7 days before bed rest (i.e., BDC-13, BDC-7); 2) two measurements on days 7 and 29 of bed rest (i.e., HDT 7, HDT 29); and 3) two measurements at 5 and 12 days post bed rest (i.e., R + 5, R + 12). Neuroimaging and behavioral assessments were conducted on the same day. The behavioral tests were administered ~3 h after neuroimaging and the testing time of day was kept as constant as possible within subjects. The functional mobility and balance tests which require the participants to be upright were only performed 2 times before best rest (i.e., BDC-13, BDC-7) and 3 times during the recovery phase: on day R + 0 (~3 h after first standing), on day R + 5, and day R + 12.

##### HDBR

The HDBR subjects completed the same measurements as the HDBR + CO_2_ subjects at 7 time points: two times pre- (i.e., BDC-12, BDC-8), three times during (i.e., HDT 8, HDT 50, HDT 65), and 2 times post- (i.e., R + 7, R + 13) HDBR. Here, we use the neuroimaging data from the HDBR study only for group comparisons with the HDBR + CO_2_ group. We examined only the *T*_1_ structural and dMRI scans from BDC-8, HDT 8, and HDT 50, as these assessment time points fell closest to those of the HDBR + CO_2_ group.

#### MRI Setting

##### HDBR + CO_2_

The participants were supplied with 0.5% CO_2_ through a mask and gas cylinder when being transported between the bed rest module and the scanner during the HDBR + CO_2_ phase. The participants were transported on an MR compatible stretcher adjusted to maintain the 6° head-down tilt position.

A ventilation mask supplying 0.5% CO_2_ was worn during the scanning session during the HDBR + CO_2_ phase, which required a small rightward head rotation to accommodate the ventilation mask tubing through the head coil. The participants wore the ventilation mask and the T-piece connector to breathe in ambient air during the baseline and re-ambulatory phases to keep the testing consistent.

The 6° HDT was maintained during MRI using a foam wedge that kept the torso and legs elevated upon transfer to the scanner table. However, the head coil and thus the head position remained horizontal.

##### HDBR

All scans were acquired in supine position in the scanner.

#### Image Acquisition

##### HDBR + CO_2_

All scans were acquired on a dedicated 3-T Siemens Biograph mMR scanner, with a 16-channel head/neck coil.

*T*_1_-weighted gradient-echo pulse scans (Magnetization Prepared Rapid Gradient Echo; MPRAGE) with the following parameters were obtained: 3D *T*_1_ sagittal overlay (TR = 1900 ms, TE = 2.44 ms, flip angle = 9°, FOV = 250 × 250 mm, slice thickness = 1.0 mm, 192 slices, matrix = 256 × 256, voxel size = 0.5 × 0.5 × 1 = 0.25 mm^3^).

Diffusion-weighted 2D echo-planar imaging scans with the following parameters were obtained: TR = 9800 ms, TE = 91 ms, flip angle = 90°, FOV = 235 × 235 mm, matrix size = 128 × 128, slice thickness = 2.7 mm, 49 axial slices with zero gap, resulting in a voxel size of 1.8 × 1.8 × 2.7 mm^3^. Thirty noncollinear gradient directions with diffusion-weighting of *b* = 1000 s/mm^2^ were sampled twice. A volume with no diffusion weighting (*b* = 0 s/mm^2^) was acquired at the beginning of each sampling stream.

##### HDBR

All scans were acquired on a 3-Tesla Siemens Magnetom Skyra MRI scanner utilizing a 32-channel head coil. The scanning parameters differed from those of the HDBR + CO_2_ group; the exact acquisition parameters are presented in the Supplementary Material (*HDBR Image Acquisition*).

#### Image Analysis

##### GM Volumetric Measures

The structural MRI data were processed using the Computational Anatomy Toolbox (CAT12 v.1363) (Gaser and Dahnke 2016) for Statistical Parametric Mapping (SPM12 version 7219) (Ashburner et al. 2014), running on MATLAB R2016a (Mathworks).

With the exception of selecting GCUT skull stripping for better results, all *T*_1_-weighted images were processed following the standard CAT12 preprocessing steps (Gaser and Kurth 2017) for longitudinal data with default parameters. This includes sample homogeneity verification, intrasubject bias correction, segmentation into Jacobian-modulated gray, white and cerebrospinal fluid (CSF) compartments, normalization to Montreal Neurological Institute (MNI) standard space with the DARTEL algorithm (Ashburner 2007) and finally, smoothing with an isotropic 8 mm full-width at half-maximum (FWHM) Gaussian kernel. We also “modulated” the images (multiplication by the Jacobian determinant derived from spatial normalization) to allow for the interpretation of GM volume effects.

The lateral, third, and fourth ventricle regions of interests (ROIs) were automatically estimated by CAT12 using the Neuromorphometrics volume-based atlas map where the CSF volume for the ROIs were estimated in native space (Gaser and Kurth 2017). Analysis of the ventricle volume change utilized the ventricle volumes collected from the second assessment time point onward, expressed as a percentage of the baseline volume. As enlargement of lateral ventricles as a result of altered CSF pathway or obstructed venous drainage is not consistently symmetrical (Bering 1962), we also evaluated asymmetry lateral ventricle changes. An asymmetry index was calculated as follows: (Left − Right)/(Left + Right) × 100.

Estimation of cerebellar volumes was conducted via CERES (CEREbellum Segmentation) (Romero et al. 2017), a patch-based multiatlas segmentation tool which automatically segments and parcellates cerebellar lobules. Given the limited contrast between gray and white matter in the cerebellum due to the subvoxel resolution of folia and arbor vitae on our scans, the total volumes for each region were utilized rather than GM measures. The analyses focused on the anterior, posterior lobes, and Crus I that are associated with sensorimotor and cognitive task performance (Bernard and Seidler 2013). ROIs were constructed by summing individual lobules (see Supplementary Table 2 for further details). As CERES calculates volumes of the lobules in native space, the cerebellar results were normalized to total cerebellum volume at the first baseline time point.

##### White Matter dMRI Indices

dMRI data were analyzed using FMRIB Software Library (FSL) version 5.0.9, MATLAB R2015b, Advanced normalization tools (ANTs 1.9.x; Avants et al. 2010; 2011) and custom written FW algorithms (Pasternak et al. 2009). Aside from the Rician noise removal for the dMRI data (Manjon et al. 2013) performed at the beginning and the reduced spatial smoothing kernel size (i.e., 3-mm FWHM Gaussian kernel) applied at the final step, we have utilized the identical dMRI processing steps previously described in Lee, Koppelmans, et al. (2019b). These additional steps were applied to filter the background noise present in the current dMRI data sets and to lessen the blurring bias, respectively. The dMRI processing steps include motion and eddy current correction of the dMRI data and production of an FW image and scalar diffusion tensor imaging indices corrected for FW for each time point and individual via an in-house developed algorithm (Pasternak et al. 2009). These images were in turn normalized to MNI152 common space utilizing ANTs’ SyN algorithm.

The FW images represent the fractional volume of the FW compartment in a voxel, indicating the proportion of water molecules that are not obstructed by surrounding cellular structures (Pasternak et al. 2009). Fractional anisotropy (FA_T_), axial and radial diffusivity (AD_T_ and RD_T_) were analyzed after FW correction; these indices are referred to with a subscript T to indicate that they are based on the tissue compartment.

#### Statistical Analysis

##### Characterization of the Impact of HDBR + CO_2_ on Brain Structure

Preprocessed GM volumes, FW, FA_T_, AD_T_, and RD_T_ images were entered into flexible factorial models (SPM’s mixed model equivalent) in order to determine brain changes across sessions. Utilizing the same approach applied in our previous bed rest studies (Koppelmans et al. 2017b; Koppelmans et al. 2017c), the change in the measures over the six sessions was evaluated using contrast vectors as weights for statistical analyses, testing for both increases and decreases. We sought to identify brain areas with either acute or cumulative changes with HDBR + CO_2_ (for details, see Supplementary Fig. 1). We tested for both increases and decreases over time in separate models, as we expected to see both effects with regional variation (as in our previous bed rest and spaceflight investigations, cf., Koppelmans et al. 2016; Koppelmans et al. 2017c). As can be seen in Supplemental Figure 1, the acute model is testing for changes that are detectable at our first in-bed rest test point (day 7) and that then remain stable. The cumulative model (panel *b*) is testing for more gradual changes over time. All contrast vectors were scaled such that the weights sum to zero.

For GM volume analyses, voxels with GM values below 0.1 were excluded (absolute threshold masking) to avoid possible edge effects around tissue borders. Analyses of diffusion indices were confined to a white matter mask except for the FW measure, for which a whole-brain mask was used. These masks were constructed following identical steps to those described in our previous study (Lee, Koppelmans, et al. 2019b). For all flexible factorial analyses, the alpha level was set at 0.05, corrected for family wise error (FWE), and the extent threshold was set to 10 voxels. There was substantial overlap between the regions showing acute and cumulative changes, differing only in cluster sizes and not locations. Thus, we present findings from the acute model unless stated otherwise.

##### HDBR versus HDBR + CO_2_

Exploratory comparisons between the HDBR and HDBR + CO_2_ groups were carried out to determine the additive effect of increased CO_2_ levels on HDT. In order to account for the different testing timelines between groups, analyses utilized the change occurring from the second BDC measure to the next two BR measures. First, we estimated the linear slope of GM volume, FW, FA_T_, AD_T_, and RD_T_ measures over these time points. Then, we divided the slope images by the intercept images (expressed in %), in order to take into account baseline differences as in our prior studies (Lee et al. 2019a; Hupfeld, Lee, et al. 2020a; Salazar et al. 2020). We entered these normalized slope images into a two sample *t*-test to examine between-group differences. We limited this group comparison to the areas where significant changes were seen as a function of HDBR + CO_2._ The ROIs were created for each measure by spatially summing all significant clusters from the acute and cumulative flexible factorial model analyses.

##### Association of Brain and Behavior Changes with HDBR + CO_2_

Recently, we reported the impact of HDBR + CO_2_ on cognition and motor performance (Lee et al. 2019a) in the sample included here. In short, in comparison to the HDBR group, the HDBR + CO_2_ group showed 1) an enhanced response consistency on the Rod and Frame Test (RFT; visual dependency assessment), 2) reduced time to complete the Digit Symbol Substitution Test (a paper-and-pencil test of processing speed), and 3) increased time to complete the Functional Mobility Test (FMT; an obstacle course partly set up on medium density foam, with hurdles and slalom pylons). Given the current objective of deciphering the additive effect of elevated CO_2_, we focused on these behavioral measures that showed differential changes during HDBR + CO_2_ relative to HDBR. An identical test battery was administered to both groups (see Koppelmans et al. 2013; Lee et al. 2019a). For each measurement, individual behavioral change scores from the BDC-7 baseline time point to the final HDBR + CO_2_ time point (HDT 29; R + 0 for the FMT only) were calculated. Similarly, the difference maps of the GM, FW, and diffusivity measures were computed using the BDC-7 and HDT 29 scans. To examine whether HDBR + CO_2_-related pre-to-post brain changes were associated with behavioral changes, general linear model (GLM) analyses were performed with the difference maps including the behavioral change score as the regressor of interest (change-change correlations). For the FMT measurement, *n* = 10 subjects were included in the analyses after removing one outlier participant. The analyses were restricted to the brain areas where significant changes were seen as a function of HDBR + CO_2._

##### SANS versus NoSANS Group Comparisons

Exploratory whole brain analyses were conducted to examine subgroup differences between the HDBR + CO_2_ subjects who developed signs of SANS (SANS; *n* = 5) and those who did not (NoSANS; *n* = 6). For each GM, FW, and diffusivity measure, two-sample *t*-tests were carried out using 1) the mean image of the two BDC scans and 2) the difference map between BDC-7 and HDT 29 scans in order to examine the SANS versus NoSANS group differences at baseline and in brain changes as a function of HDBR + CO_2_, respectively. Finally, an examination was conducted to identify brain areas where differential brain-behavior associations might occur between the groups. We focused only on the measures in which we previously observed significant subgroup differences (Lee et al. 2019a). The measures include 1) the RFT frame effect and response consistency measure, 2) finger tapping accuracy under both single (finger tap only) and dual task conditions (finger tap while keeping count of an oddball target stimulus), 3) finger tapping reaction time (RT) under the dual task condition, and 4) the equilibrium score of the eyes open, head erect posture condition (EO), measuring postural stability while standing on a foam pad. GLM analyses were performed with the difference maps between BDC-7 and HDT 29 of each brain measure and the regressor of interest was the subgroup status multiplied by the demeaned change in performance scores between BDC-7 and HDT + 29 (and R + 0 for the posturography test only).

Analyses were carried out using a nonparametric permutation test using a threshold-free cluster enhancement (Smith and Nichols 2009) approach with 15 000 random permutations implemented in FSL’s randomize (Winkler et al. 2014). Identical masks utilized in the flexible factorial models were applied when the analyses were not confined to certain ROIs. Variance smoothing of 2.5-mm FWHM was applied and analyses were adjusted for multiple comparisons by applying a voxel level FWE correction (*P* < 0.05) (Smith and Nichols 2009).

Linear mixed model analyses were used to examine group × time differences between 1) HDBR + CO_2_ and HDBR subjects and 2) SANS and NoSANS subjects in the ventricular volumes, lateral ventricle asymmetry index, and cerebellar volumes. As the intervals between test dates and the overall study duration differed for the 2 studies, time was entered as a continuous variable for the group comparison. Restricted maximum likelihood was selected in all linear mixed model analyses for its greater robustness to small sample bias than traditional maximum likelihood (Van Dongen 1999). Alpha levels were set at 0.05 for all analyses. SPSS21 (Disbrow et al. 2001) was used for the ROI statistical analyses.

For GM analyses utilizing modulated normalized images, individual differences in brain size were corrected by entering total intracranial volume (TIV) in the model as a covariate. Age, sex, and exercise group status were entered as covariates of no interest in all HDBR + CO_2_ versus HDBR group comparison analyses. All within HDBR + CO_2_ group analyses and SANS subgroup analyses were controlled for age and sex.

**Figure 1:**
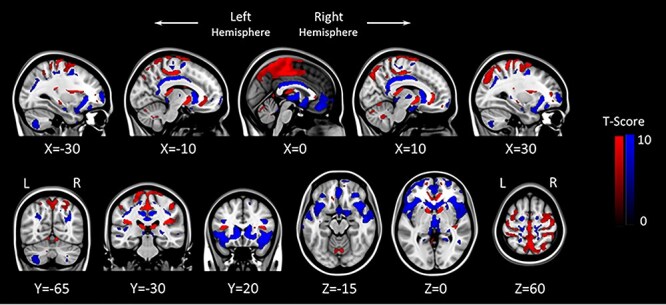
GM flexible factorial model results (*P* = 0.05, FWE corrected). Areas showing acute GM increase with HDBR + CO_2_ are marked in red, and brain regions showing acute GM decrease with HDBR + CO_2_ are marked in blue. The left side of the coronal and axial images (bottom row) corresponds with the left hemisphere of the brain. L: Left, R: Right.

### Results

#### Effects of HDBR + CO_2_ on Brain Structure

**Figure 2:**
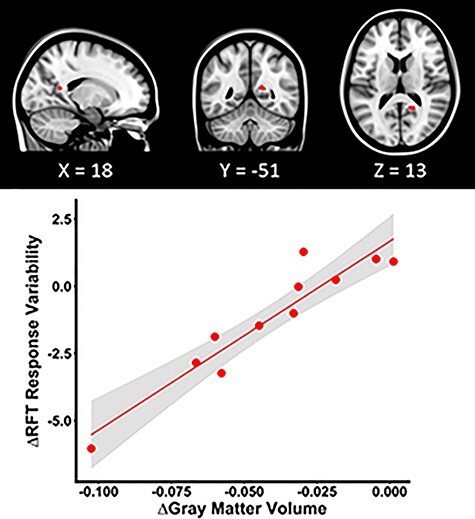
GM structure and function correlation within the HDRB + CO_2_ group. HDRB + CO_2_ individuals who showed more variable RFT response patterns at HDT29 than at BDC-7 exhibited less reduction in the precuneus cortex volume, marked in red, at HDT29 than at BDC-7. The scatter plot depicts the pre-to-post bed rest mean change in GM volume of the peak voxel as a function of the pre-to-post bed rest change in RFT response variability. The shaded area indicates the 95% confidence interval.

A paired samples *t*-test revealed no significant change in arterial PaCO2 pressure from pre-to-post bed rest (*P* > 0.05). Flexible factorial model analyses revealed extensive changes in GM volume following our a priori hypotheses. These changes include increased GM volume in the posterior aspect of the vertex and decreased volume at the base of the cerebrum (Fig. 1). Post hoc analysis revealed that these GM volume changes were not fully recovered to the BDC-7 baseline level by R + 12 (Supplementary Fig. 2*a*). Structure–function analyses revealed that those who showed less decrements in precuneus cortex volumes at HDBR + CO_2_ day 29 than BDC-7 had more variable RFT response patterns with bed rest (see Fig. 2). Likewise, those that exhibited greater volume decreases had less variable RFT responses.

#### Effects of HDBR + CO_2_ versus HDBR on GM Volume

**Figure 3:**
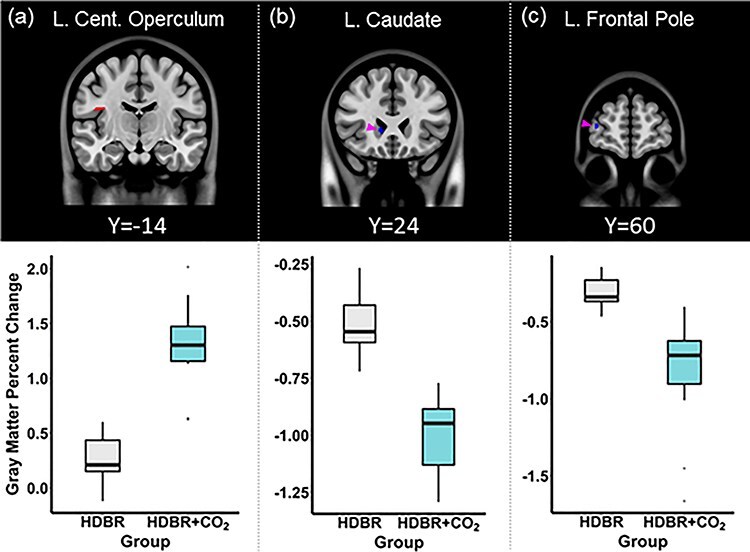
HDBR versus HDBR + CO_2_ structural brain change. The top panel depicts the brain areas in which significant (*P* = 0.05, FWE corrected) group differences in the degree of GM volume change were observed. The HDBR + CO_2_ group showed greater GM volume increase in (*a*) the left central operculum cortex and greater decrease in (*b*) the left caudate (highlighted by the pink arrow) and (*c*) left frontal pole (highlighted by the pink arrow), in comparison to the HDBR group. The bottom panel illustrates the average degree of GM volume change within the peak voxel of the clusters shown above, in the HDBR and HDBR + CO_2_ groups. L: Left, Cent: Central.

While some metrics and brain regions exhibited significant intercept differences between the two cohorts, there were more effects associated with the intervention. Group comparisons revealed that the HDBR + CO_2_ group exhibited greater left parietal lobe GM volume increases and greater left caudate and frontal pole volume decreases in comparison to the HDBR group (Fig. 3*a*–*c*). A significant group by time interaction effect was observed for the third ventricle volume; where the HDBR + CO_2_ group showed significantly greater increments in the third ventricle volume (% baseline measurement) during bed rest in comparison to the HDBR group (group × time *β* = −0.208, *P* = 0.049, group *β* = 6.041, *P* = 0.37, time *β* = 0.638, *P* < 0.01, Supplementary Fig. 3*a*). There were no significant group by time interaction effects in the lateral ventricle volumes, fourth ventricle volumes, and the lateral ventricle asymmetry changes. The HDBR + CO_2_ group also showed a significant increase in cerebellar Crus I volume (% baseline total cerebellum volume) during bed rest relative to the HDBR group (group × time *β* = −0.013, *P* < 0.01, group *β* = −0.244, *P* = 0.88, time *β* = 0.015, *P* < 0.01, Supplementary Fig. 3*b*). Post hoc analyses revealed no significant group difference in any of the ventricle measures, lateral ventricle asymmetry index, and total cerebellum volume at baseline (i.e., first time point measurement).

#### Effects of HDBR + CO_2_ on FW

**Figure 4:**
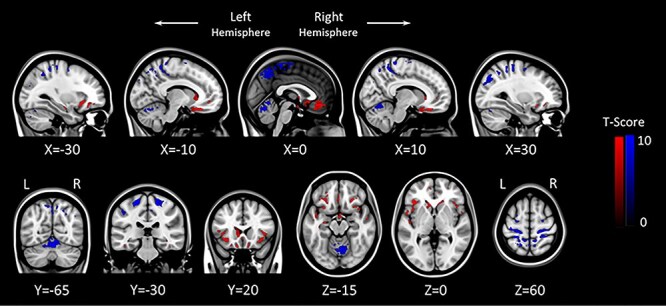
FW flexible factorial model analysis results (*P* = 0.05, FWE corrected). Areas showing acute FW increase with HDBR + CO_2_ are marked in red. Inversely, brain regions showing acute FW decrease with HDBR + CO_2_ are marked in blue. The left side of the coronal and sagittal images (bottom row) corresponds to the left hemisphere of the brain. L: Left, R: Right.

We observed widespread FW changes with the HDBR + CO_2_ intervention. There were significant FW increases over time along the base of the frontal and temporal lobes and decreases in the posterior aspect of the cerebral vertex and the dorsal cerebellum (Fig. 4). Post hoc analyses indicate that these FW changes did not return to the BDC-7 level on R + 12 (Supplementary Fig. 2*b*). There were no significant associations between changes in FW and changes in behavioral performance as a result of the HDBR + CO_2_ intervention.

#### Effects of HDBR + CO_2_ versus HDBR on FW

**Figure 5:**
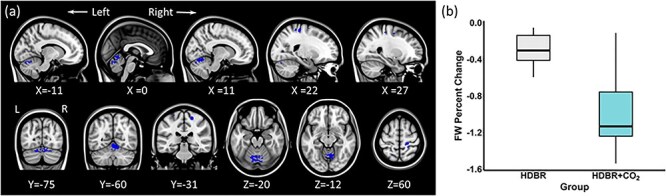
HDBR versus HDBR + CO_2_ FW change. (*a*) In comparison to the HDBR group, the HDBR + CO_2_ group showed greater FW decreases in the areas marked in blue (*P* = 0.05, FWE corrected). (*b*) The average FW percentage change in the peak voxel of the largest cluster (Cerebellum: MNI coordinate 11, −62, −17) in the HDBR and HDBR + CO_2_ groups. The left side of the image corresponds with the left hemisphere of the brain. L: Left, R: Right.

In comparison to the HDBR group, the HDBR + CO_2_ group showed significantly greater FW reductions in the pre- and post-central gyri, angular gyrus, lateral occipital cortex, and the cerebellum (Fig. 5). There were no brain areas showing significantly greater FW increases with HDBR + CO_2_ compared with HDBR alone.

#### Effects of HDBR + CO_2_ on White Matter Microstructure

**Figure 6:**
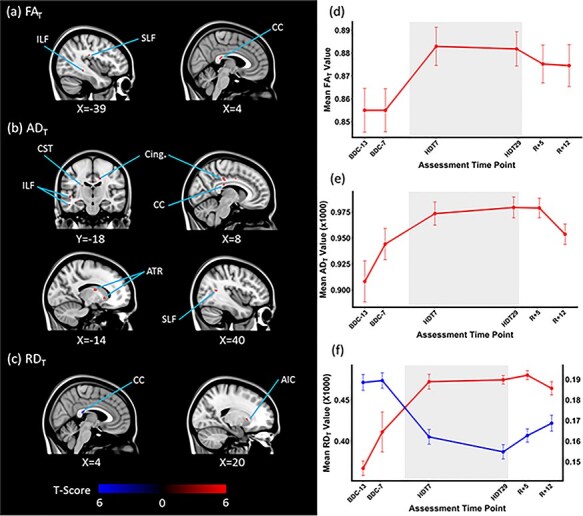
HDBR + CO_2_ effects on white matter diffusivity. White matter structures showing significant acute changes in (*a*) FA_T,_ (*b*) AD_T_, and (*c*) RD_T_ values as a function of HDBR + CO_2_ are depicted. Areas showing positive changes are marked in red and negative changes are marked in blue (*P* = 0.05, FWE corrected). The average (*d*) FA_T_, (*e*) AD_T_, and (*f*) RD_T_ values of each assessment time point of the peak voxel of the largest cluster showing acute changes with HDBR + CO_2_ are shown. (*a*) The acute increase in FA_T_ value of the splenium of the corpus callosum (MNI 4, −32, 17) and (*b*) the acute increase in AD_T_ values of the Body of corpus callosum (MNI 16, −18, 36). (*f*) The acute increase in RD_T_ value of the right anterior limb of internal capsule (MNI 20, 14, 1) in red and the acute decrease in RD_T_ value of the body of corpus callosum (MNI 3, −30, −18) in blue. The axis of the acute negative change is shown on the right side. The bed rest phase is demarcated in gray. The error bars indicate SEM. ILF: Inferior longitudinal fasciculus, SLF: Superior longitudinal fasciculus, CC: Corpus callosum, CST: Corticospinal tract, Cing.: Cingulate, ATR: Anterior thalamic radiation, AIC: anterior limb of the internal capsule.

As a function of HDBR + CO_2_, both FA_T_ and AD_T_ values showed significant increases in white matter structures including the superior and inferior longitudinal fasciculi, inferior fronto-occipital fasciculus, and corpus callosum. Increased AD_T_ measures were also observed in the anterior thalamic radiation and the corticospinal tract. An increase in RD_T_ was observed in the right anterior limb of the internal capsule; decreased RD_T_ values were predominantly observed in the body of the corpus callosum (Fig. 6). Supplementary Table 3 lists the clusters in which significant acute changes in the diffusivity indices with HDBR + CO_2_ were observed. Regression analyses revealed no significant associations between white matter diffusivity changes and behavioral performance changes as a result of the HDBR + CO_2_ intervention.

#### Effects of HDBR + CO_2_ versus HDBR on White Matter Microstructure

**Figure 7:**
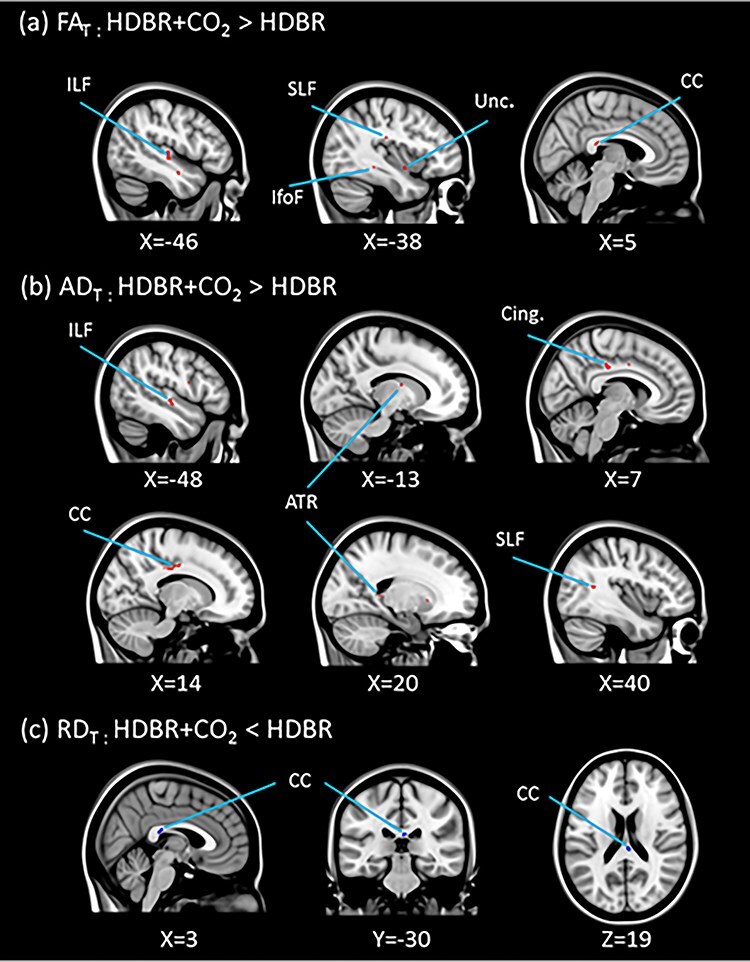
HDBR versus HDBR + CO_2_ on white matter diffusivity. In comparison to the HDBR group, the HDBR + CO_2_ group showed greater (*a*) FA_T_ and (*b*) AD_T_ increases in the areas marked in red, and greater (*c*) RD_T_ decreases in the areas marked in blue (*P* = 0.05, FWE corrected). ILF: Inferior longitudinal fasciculus, SLF: Superior longitudinal fasciculus, IfofF: Inferior fronto-occipital fasciculus, Unc.: Uncinate fasciculus, CC: Corpus callosum, Cing.: Cingulate, ATR: Anterior thalamic radiation.

In comparison to the HDBR group, the HDBR + CO_2_ group showed significantly greater FA_T_ increases in white matter structures including the body of the corpus callosum, the superior and inferior longitudinal fasciculi, and the left uncinate fasciculus. AD_T_ values increased in these white matter structures to a greater extent in the HDBR + CO_2_ group as well. Additionally, greater increases in AD_T_ values in the right cingulum and the left anterior thalamic radiation were observed in the HDBR + CO_2_ group in comparison to the HDBR group. In contrast, greater RD_T_ decreases were observed in the HDBR + CO_2_ group than the HDBR group in the body of the corpus callosum (Fig. 7). The clusters in which the degree of the white matter diffusivity change was significantly different between the two groups are listed in Supplementary Table 4.

#### SANS versus NoSANS

The SANS and NoSANS groups did not exhibit significant differences in any of the brain structural or dMRI measures at baseline. In addition, there were no differential effects of HDBR + CO_2_ on the SANS subgroups; the two groups did not differ in the pre-to-post bed rest change in whole brain GM, ventricle and cerebellum volumes, or any of the dMRI measures. After removing an outlying HDT 29 time point measure of one NoSANS individual to achieve normal distribution of the residuals meeting the linear mixed model assumption, the lateral ventricle asymmetry scores showed differential changes between the SANS subgroups (group × time *β* = 0.613, *P* = 0.02, group *β* = −3.476, *P* = 0.81, time *β* = −0.393, *P* = 0.03, Supplementary Fig. 4) The SANS group showed a right-biased enlargement of the lateral ventricles, and the NoSANS group exhibited a left-biased asymmetrical enlargement as a function of HDBR + CO_2_. The result was replicated even after removing all data points of this outlying subject.

**Figure 8:**
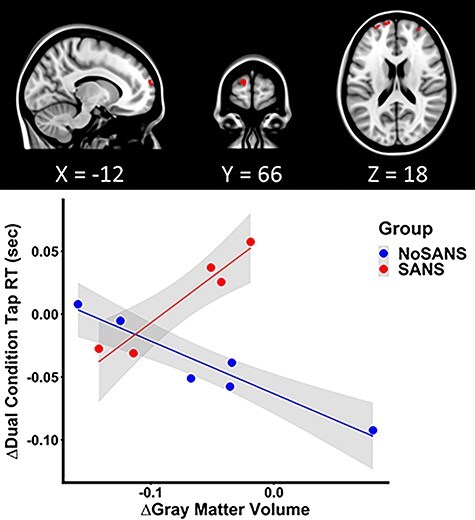
Differential GM structure and function association between the SANS and NoSANS groups. The SANS group individuals who showed greater increases in the frontal pole volume from BDC-7 to HDT 29 exhibited greater slowing of dual condition finger tap RTs, while the NoSANS group showed the contrasting brain-behavioral association pattern. The scatter plot depicts the pre-to-post bed rest mean change in GM volume of the peak voxel of the largest left frontal pole cluster (MNI coordinate: −12, 66, 18) as a function of the pre-to-post bed rest change in dual condition finger tap RTs. The shaded areas indicate the 95% confidence interval.

Regression analyses revealed that the SANS and NoSANS subgroups had differential associations between change in frontal pole GM volume and change in dual task finger tapping RT as a function of HDBR + CO_2_. Specifically, in the SANS group, increased frontal pole volume from pre- to post-bed rest was associated with greater slowing of dual task finger tapping RT following the intervention, while the NoSANS group showed the opposite effect (Fig. 8).

### Discussion

In the current study, we investigated brain volumetric and white matter mictrostructural consequences of exposure to simulated microgravity combined with mild hypercapnic conditions. In line with previous spaceflight analog studies (Li et al. 2015; Roberts et al. 2015; Koppelmans et al. 2017a; Koppelmans et al. 2017c), we found GM volumetric increases in posterior-parietal areas and decreases in fronto-temporal regions, and FW increases at the base of the cerebrum and decreases along the posterior vertex. These changes partially resolved during the recovery phase. The coinciding FW increases and decreases suggest a fluid redistribution within the cranium. In particular, the ventral pooling of FW lends support to the notion that the brain is globally shifted upward. Moreover, the concomitant reduction in FW in the posterior-parietal areas indicates that the increased GM volume in these regions reflect crowding of the brain parenchyma around the vertex. Importantly, this brain positional displacement and complementing intracranial fluid shift have been observed and replicated in multiple studies in astronauts (Koppelmans et al. 2016; Roberts et al. 2017; Lee, Koppelmans, et al. 2019b) and cosmonauts, including a comparison to time-matched controls (Van Ombergen et al. 2018). The reproducibility of the spaceflight-associated brain morphological changes in ground-based analog studies suggests that these global brain changes are driven by mechanical effects (i.e., reduced load along the body longitudinal axis in microgravity and reorientation of the head relative to the gravitational vector in HDBR).

In comparison to the HDBR group, the HDBR + CO_2_ group showed greater enlargement of the left operculum cortex. Despite the lack of observed increase in P_a_CO_2_ with the HDBR + CO_2_ intervention, factors influenced by P_a_CO_2_ changes such as cerebral perfusion (Reivich 1964) could be a candidate mechanism underlying the brain changes with the additional elevated CO_2_. Another possibility is that this finding reflects an adaptive sensory reweighting process induced by group differences in activity during HDBR. The operculum cortex, adjacent to the insula body representation and the vestibular cortex, is considered a secondary somatosensory cortex with somatosensory representations of the fingers and foot (Ruben et al. 2001). Although statistically adjusted for, the HDBR + CO_2_ group were physically inactive, while the majority of the HDBR group participated in an exercise intervention. This reduced level of sensory input may have led to a relatively greater compensatory enlargement of the cortical representation. In contrast, the HDBR + CO_2_ group exhibited greater volumetric decrements in the left caudate and frontal pole than the HDBR group. When considering the positional shift of the brain, these GM morphological changes may reflect the influence of increased FW in the nearby structures. HDBR may result in expansion of frontal lobe sulci and the lateral ventricles, which are in proximity of the caudate nucleus (Roberts et al. 2015). The greater degree of FW reduction with HDBR + CO_2_ in dorsal aspects of the brain may suggest greater FW accumulation along the ventral side, although not to the extent of being statistically significant. Alternatively, fMRI studies combined with heart rate variability analyses or in hypercapnia have shown the involvement of basal ganglia areas such as the caudate in autonomic (Napadow et al. 2008) and respiratory (Harper et al. 2005) function. Similarly, elevated CO_2_ has been shown to selectively favor increased frontal lobe perfusion (Tchistiakova et al. 2013; Bhogal et al. 2015). Thus, the reduced GM volume could reflect the effects of sustained autonomic or cardiovascular system deconditioning that occur with HDBR interventions (Schmedtje Jr. et al. 1996). Nonetheless, given the caudate nucleus’s involvement in motor, cognitive, and emotion control (Di Martino et al. 2008), and the frontal pole functionality enabling cognitive flexibility (for review, see Koechlin 2011), the greater GM volume reduction in these areas is noteworthy.

We observed a greater degree of FW reduction along the dorsal and posterior portion of both the cerebrum and the cerebellum in the HDBR + CO_2_ group compared with the HDBR group. This could account for the greater enlargement of the cerebellar Crus I volume observed in the HDBR + CO_2_ group than in the HDBR group. Furthermore, the third ventricle expanded at a greater degree with HDBR + CO_2_ than with HDBR alone as well. In previous HDBR studies, assessment of ventricle CSF volume changes was either not a research focus or was limited due to high individual variability (Di Martino et al. 2008; Roberts et al. 2015). However, increased CSF volume in the lateral and the third supratentorial ventricular structures with spaceflight has been reported (Roberts et al. 2017; Van Ombergen et al. 2019). In line with these findings, obstruction of CSF resorption structures such as the superior sagittal sinus and arachnoid granulations due to the upward brain shift would most likely account for the third ventricle enlargement observed in the current study. Although it is difficult to determine whether the additional 0.5% CO_2_ facilitated the FW expulsion, together the FW and the third ventricle findings suggest that a greater degree of intracranial fluid shift occurred in the HDBR + CO_2_ group_._

Individuals who showed less decrease in precuneus cortex volume as a result of HDBR + CO_2_ had greater RFT response variability at the end of HDBR + CO_2_ than at baseline. The right precuneus cortex, a portion of the superior parietal cortex, has been shown to be essential in processing visual contextual cues. In particular, activation of this area plays a role in inhibiting interfering information to form and maintain the egocentric reference frame of gravitational vertical (Walter and Dassonville 2008; 2011; Lester and Dassonville 2014). Interestingly, our results suggest that enlargement of precuneus GM may lead to reduced performance. However, it is important to note that on *T*_1_-weighted MRI scans, inflow of CSF into the interstitial fluid could show as increased GM volume, and outflow as decreased GM volume. Therefore in the context of head down tilt bed rest, where brain edema could be induced (Caprihan et al. 1999) or increased CSF pressure could lead to CSF infiltration into extracellular space (Page 1985), interpretation of GM enlargement should be made with caution. Although enlargement of the precuneus cortex with 30 days of HDBR has been previously reported (Li et al. 2015), we now provide evidence that precuneus volume change as a function of HDBR is related to the variability in vertical perception in the presence of a tilted frame of reference.

We observed white matter microstructural changes throughout the whole brain as a function of HDBR + CO_2_. The white matter changes predominantly, although not exclusively, manifested as increased FA_T_ and AD_T_ values that trended back toward baseline values during the recovery phase. In the context of prolonged HDBR, the viscoelastic white matter tracts would conform to the global morphological change of the brain parenchyma. Indeed, white matter structures that are aligned along the medio-lateral and antero-posterior axis exhibited increased diffusivity along the longitudinal axis. The corpus callosum, lying posterior to the lateral ventricles, and the inferior longitudinal fasciculus showed the largest changes. Therefore, it is most likely that the increased FA_T_ and AD_T_ values reflect the partial volume changes, representing the condensing and “straightening” of undulating fibers (Nilsson et al. 2012) occurring with the global brain and fluid shifts and change in physical pressure. Conversely, increased RD_T_ values were observed in the anterior limb of the right internal capsule. This white matter tract runs between the caudate and the lentiform nucleus, areas that exhibited reduced volumes following HDBR + CO_2_. The increased radial diffusivity or diffusion in the direction perpendicular to the main longitudinal axis may reflect unpacking of the white matter fiber bundles resulting from morphological changes of the surrounding subcortical structures. This pathway connects the thalamus and cerebellar pontine tracts with the prefrontal cortex, so the changes may also reflect declines resulting from decreased sensory inputs in the bed rest context. It is also possible that the deterioration of nonparallel fibers present in the voxel (Jeurissen et al. 2013), to manifest as increased FA_T_ and AD_T_ values mentioned above. Future studies using higher spatial resolution scans and analytic approaches such as constrained spherical deconvolution will help disambiguate these findings.

In a prior HDBR study, increased FA values in overlapping regions as the current study have been reported (Li et al. 2015). However, no corrections for multiple comparisons were made in this prior study. In our previous HDBR study in which we did employ such corrections, we did not observe white matter changes (Koppelmans et al. 2017c). The smaller smoothing kernel utilized in the current study may have increased the sensitivity to detect microstructural changes in thinner tracts. Moreover, we found greater FA_T_ and AD_T_ increases and RD_T_ decreases in the HDBR + CO_2_ group than in the HDBR group. This is particularly intriguing given our recent observation of decreasing FA_T_ and AD_T_ values and increasing RD_T_ values in similar white matter tracts in astronauts (Lee, Koppelmans, et al. 2019b). The contradicting white matter results between spaceflight and the current study supports the notion that a unique factor of spaceflight is at play driving the brain white matter microstructural changes. However, it may also be that we would see comparable white matter changes to those reported with spaceflight in a longer duration bed rest study. Future studies with a larger sample size or extended duration of HDBR + CO_2_ may help determine the exact mechanism underlying the increases in anisotropic white matter diffusivity and identify the functional consequence or the limit of the adaptive responses of the brain white matter until potential disruption would occur.

None of the structural or fluid brain changes reported here correlated with the performance on the digit symbol substitution task. We previously reported that the participants in this study exhibited a greater facilitation of performance on this task during HDBR + CO_2_ than those in our study of HDBR in ambient air, similar to a report of enhanced cognition with 26 h of HDBR combined with 0.5% CO_2_ (Basner et al. 2018). While anecdotal astronaut reports of “space fog” have been made, there is only little evidence to support cognitive changes with spaceflight (Strangman et al. 2014). There is evidence that dual tasking performance is negatively affected in microgravity (Manzey et al. 2000) and NASA’s recent “twins” study also showed an increase in risky behavior in a cognitive task, as well as decreased accuracy in a visual object learning task, in one individual during flight (Garrett-Bakelman et al. 2019). Further work is required to better understand the impact of brain changes with spaceflight on operational performance and to ascertain whether brain changes reflect adaptation or dysfunction (Hupfeld et al. 2021).

We also conducted exploratory stratified analyses of SANS versus NoSANS individuals, in order to identify the brain morphological characteristics of those who developed symptoms of SANS in a ground-based spaceflight analog model. Optic disc edema in astronauts, one symptom of SANS, occurs asymmetrically, with a predisposition for the right side (Mader et al. 2017). It has been hypothesized by Aintablian et al. (2018) that the ramification of a cephalad fluid shift induced obstruction of venous and lymphatic mechanisms would be particularly pronounced on the right side, given the anatomical trait of predominant right side CSF drainage (Scotti et al. 1988; Ayanzen et al. 2000). In spaceflight, ventricular volume expansion has been considered as a compensatory mechanism (Van Ombergen et al. 2019; Wostyn et al. 2020) demonstrated by reduced ventricular volume expansion in astronauts with SANS symptoms (Roberts et al. 2019). Although it is difficult to conclude whether the right-biased enlargement of the lateral ventricle seen in the SANS group here reflects asymmetrically sequestered CSF due to the increased strain of right dominant CSF drainage, the finding may aid in further understanding of the etiological mechanisms of SANS. Conditions that occur on Earth and have been suggested to be similar to SANS include those with low folate status (these individuals are more susceptible to SANS in spaceflight (Zwart et al. 2016) and normal pressure hydrocephalus. Investigation of similar measures in these populations may enhance our understanding of SANS and potentially improve treatment of these conditions on Earth.

We observed differential frontal pole volumetric change associations, where the SANS group showed slowing and the NoSANS group showed the facilitation of finger tap RT under dual task conditions. Frontal lobe areas including the frontal pole have been considered as the central executive structures involved in coordination of subsidiary functional networks or to contribute to the working memory capacity necessary for dual task performance (Salmon et al. 1996). The results may reflect a disparity of the innate functional architecture between the two subgroups that culminated in a differential structure–function association, a likelihood that would need to be investigated in conjunction with future fMRI studies. The differential functional sequella of the SANS subgroups calls for further characterization and holds important implications for crewmember performance, should the findings hold in a larger sample. Given the small subgroup sample sizes here, these SANS versus NoSANS differences should be interpreted with caution.

The small sample size of the present study precludes the generalizability of findings. Additionally, inclusion of an ambulatory control group and an HDBR group without CO_2_ enrichment, but with matching scanning timelines and parameters and tested on the same scanner as the HDBR + CO_2_ group, would further aid in examining the additive effect of CO_2_ enrichment. The HDBR + CO_2_ and HDBR groups were each part of separate bed rest campaigns, in which the study design was restricted by NASA. However, as the analyses were all longitudinal in nature, the measures were inherently normalized to each individual. While this does not eliminate differences between the campaigns, this suggests that our findings are less susceptible to differences in study design. That is, each of the studies was meant to stand on their own, with subjects serving as their own controls. Thus, our statistical contrasts searched for brain regions where our metrics were stable across the first two pre-intervention time points and that then increased (or decreased) throughout the course of the intervention, with a subsequent reversal toward pre-intervention levels following bed rest completion (as illustrated in Supplemental Fig. 1). It is highly unlikely that scanner effects such as drift, software upgrades, and hardware upgrades would result in this precise pattern of temporal changes, but future studies would certainly benefit from time-matched controls. Although preliminary in nature, the compelling findings presented here are an important first step in understanding the influence of spaceflight factors namely, the combined effect of 0.5% CO_2_ and headward fluid shifts (Berry et al. 1966; Moore and Thornton 1987; Nelson et al. 2014) on the human brain and behavior.

## Notes

The authors gratefully acknowledge the VaPER study staff for their support throughout the duration of the project. *Conflict of Interest*: None declared.

## Funding

NASA (#80NSSC17K0021, NSBRI SA02802).

## Supplementary Material

Supplementary_Data
