# Supplementary material for: Effects of Spaceflight Stressors on Brain Volume, Microstructure, and Intracranial Fluid Distribution
Source: Cereb Cortex Commun. 2021 Mar 30;2(2):tgab022. doi: 10.1093/texcom/tgab022 (PMC8152913; doi:10.1093/texcom/tgab022)

Supplementary Materials

Methods

HDBR Image Acquisition

T1 structural scans: T1-weighted gradient-echo pulse scans with the following parameters were obtained: 3D T1 sagittal overlay (TR = 1900 ms, TE = 2.49 ms, flip angle = 9°, FOV = 270×270 mm, slice thickness = 0.9 mm, 192 slices, matrix = 288×288, voxel size = 0.94×0.94×0.90 = 0.80 mm^3).

dMRI scans: diffusion-weighted 2D echo-planar imaging scans with the following parameters were obtained : TR = 10100 ms, TE = 95 ms, flip angle = 90°, FOV = 240 × 240 mm, matrix size = 128 × 128, slice thickness = 2mm, 75 axial slices with zero gap, resulting in a voxel size of 1.88 × 1.88 × 2 mm. Other parameters were constant with the HDBR+CO_2 protocol.

In five of our 15 subjects, shorter TE (2.44-2.46) and smaller FOVs were inadvertently applied when obtaining the T1 scans, altering the voxel resolution from 0.94mm to 0.98mm. Two subjects out of the 15 had no HDT 8 time point dMRI data. In 5 dMRI scans the scanning parameters were unintentionally altered. The alterations include additional slices collection (80-87) in 3 scans with increased TR (10700-12000) and altered FOV resulting in a change in voxel resolution in one scan (1.88 mm to 1.95 mm). In 2 other dMRI scans the increased TR (10200) and reduced FOV resulted in a voxel resolution of 1.72 mm. The alterations were minor and were not systematically distributed over the baseline and intervention time points.

Results

The peak voxels within clusters showing GM and FW changes were labeled using the Harvard-Oxford Cortical Structural Atlas. Anatomical labels of peak voxels showing significant white matter changes were defined using the JHU white-matter tractography atlas. If no labels were detected, the ICBM-DTI-81 white-matter labels atlas and the Harvard-Oxford Cortical Structural Atlas were consulted.

Supplementary Table 1. Demographic Information. Body mass index (BMI), height, weight and age were recorded at the start of the study.

	HDBR (M=15)		HDBR+CO_2 (F=5, M=6)	
Age (years)	29.9 (3.9)		33.9 (8.0)	
Height (cm)	177.9 (7.6)		173.8 (9.8)	
Weight (kg)	77.5 (11.0)		70.8 (8.6)	
BMI (kg/m^2)	24.4 (2.7)		23.4 (2.2)	
	Control (M=3)	Exercise (M=12)	SANS (F=3, M=2)	noSANS (F=2, M=4)
Age (years)	31.7 (5.9)	29.4 (3.4)	37.8 (7.5)	30.7 (7.5)
Height (cm)	178.0 (7.9)	177.9 (7.9)	169.6 (8.2)	177.4 (10.4)
Weight (kg)	84.2(6.0)	75.8 (11.5)	71.5 (8.3)	70.2 (9.5)
BMI (kg/m^2)	26.6 (0.9)	23.9 (2.7)	24.8 (2.0)	22.3 (1.8)

HDBR: Head Down Bed Rest, F: Females, M: Males
SANS: Spaceflight Associated Neuro-ocular Syndrome
All data are presented as mean (SD).

Supplementary Table 2. List of CERES lobules in each cerebellar ROIs. The bilateral volume (in cm^3) of each lobule were combined for the analyses.

**Cerebellum ROI**	**CERES Atlas labels**
Anterior Lobe	Lobule I-II
	Lobule III
	Lobule IV
	Lobule V
	Lobule VI
Posterior Lobe	Lobule VIIb
	Lobule VIIIa
	Lobule VIIIb
	Lobule IX
	Lobule X
	Lobule Crus II
Crus I	Lobule Crus I

Supplementary Table 3. Acute changes in white matter diffusivity with HDBR+CO_2. Cluster size, the *T*- value, MNI coordinates and the label of the peak voxel of the clusters showing significant acute changes in FA_T, AD_T and RD_T with HDBR+CO_2 are listed. While FA_T and AD_T showed significant increases with HDBR+CO_2, RD_T exhibited both significant increase and decreases with HDBR+CO_2 at $P<0.05$ (FWE corrected). L: Left R: Right

	Cluster	Voxels	T-value	X (mm)	Y (mm)	Z (mm)	
**FA_T**							
	1	62	7.06	4	-32	17	Splenium of corpus callosum
	2	25	6.82	-36	-15	-13	Inferior fronto-occipital fasciculus L, Anterior thalamic radiation L
**Acute Positive**	3	22	7.18	-39	-36	-9	Inferior fronto-occipital fasciculus L, Inferior longitudinal fasciculus L
	4	13	6.83	13	-37	9	Splenium of corpus callosum
	5	12	7.17	-39	-22	24	Superior longitudinal fasciculus L
**AD_T**							
	1	142	7.10	16	-18	36	Body of corpus callosum
	2	107	7.53	-49	-16	-6	Inferior longitudinal fasciculus L
	3	103	7.46	-15	-6	15	Anterior thalamic radiation L
	4	89	6.62	19	13	3	Anterior limb of internal capsule R
	5	61	6.46	-49	-19	-21	Inferior longitudinal fasciculus L
	6	48	6.38	3	-29	20	Body of corpus callosum
**Acute Positive**	7	46	7.06	8	-27	24	Body of corpus callosum
	8	29	6.58	27	-49	21	Inferior fronto-occipital fasciculus R
	9	27	6.63	39	-50	13	Superior longitudinal fasciculus R
	10	23	6.80	-1	4	23	Body of corpus callosum
	11	20	6.41	23	-7	22	Anterior thalamic radiation R
	12	19	6.34	45	-29	32	Superior longitudinal fasciculus R
	13	14	6.55	-22	-18	20	Corticospinal tract L
	14	12	6.46	-48	4	18	Superior longitudinal fasciculus L
**RD_T**							
**Acute positive**	1	20	6.27	20	14	1	Anterior limb of internal capsule R
	1	101	8.16	3	-30	18	Body of corpus callosum

**Acute negative**	2	18	6.47	39	-2	-34	Inferior longitudinal fasciculus R
	3	11	6.24	-39	-35	-8	Inferior fronto-occipital fasciculus L
	4	10	6.24	37	-40	-5	Inferior fronto-occipital fasciculus R

Supplementary Table 4. List of clusters showing significant HDBR vs. HDBR+CO_2 group differences in white matter diffusivity change. Along with the size of the cluster, the *P* value, MNI coordinates and the label of the peak voxel of the cluster is listed. In comparison to the HDBR group, the HDBR+CO_2 group showed significantly greater increase in the FA_T and AD_T values and significantly greater decrease in the RD_T values at P<0.05 (FWE corrected).

	Cluster	Voxels	*P* (FWE corr.)	X (mm)	Y (mm)	Z (mm)	
**FA_T**							
**HDBR < HDBR+CO2**	1	118	<0.001	-46	-18	1	Inferior longitudinal fasciculus L
	2	50	0.009	5	-30	19	Body of corpus callosum
	3	46	<0.001	-44	-6	-17	Inferior longitudinal fasciculus L, Uncinate fasciculus L, Inferior fronto-occipital fasciculus L
	4	28	0.001	-38	-22	24	Superior longitudinal fasciculus L, Superior longitudinal fasciculus (temporal part) L
	5	19	0.009	-38	-36	-10	Inferior longitudinal fasciculus L, Inferior fronto-occipital fasciculus L
	6	12	0.038	50	-19	-27	Superior longitudinal fasciculus (temporal part) R, Superior longitudinal fasciculus R
	7	12	0.004	-38	-2	-8	Uncinate fasciculus L, Inferior fronto-occipital fasciculus L
	8	10	0.030	49	-22	-17	Inferior longitudinal fasciculus R
**AD_T**							
**HDBR < HDBR+CO2**	1	120	0.004	11	-16	31	Body of corpus callosum
	2	91	0.012	-47	-14	-7	Inferior longitudinal fasciculus L
	3	86	<0.001	7	-27	34	Cingulum (cingulate gyrus) R
	4	17	0.015	40	-51	12	Superior longitudinal fasciculus R, Superior longitudinal fasciculus (temporal part) R
	5	13	0.001	7	-2	37	Cingulum (cingulate gyrus) R, Cingulum (cingulate gyrus) L
	6	12	0.021	-12	-4	14	Anterior thalamic radiation L
	7	11	0.038	20	15	0	Inferior fronto-occipital fasciculus R, Anterior limb of internal capsule R
**RD_T**							
**HDBR > HDBR+CO2**	1	72	0.003	4	-28	21	Body of corpus callosum

Supplementary Figure 1. Contrast weights used to examine the time course of brain changes with HDBR+CO_2. The contrast model in (a) assumes a stable baseline, acute change in brain measures during bed rest followed by a full recovery occurring post bed rest. The contrast model in (b) assumes a stable baseline, cumulative change in brain measures during bed rest and gradual full recovery following bed rest. Red and orange lines depict the positive form of each contrast; blue and purple lines depict the negative form of each contrast model. BDC: baseline data collection; HDT: head-down tilt, R: Recovery.

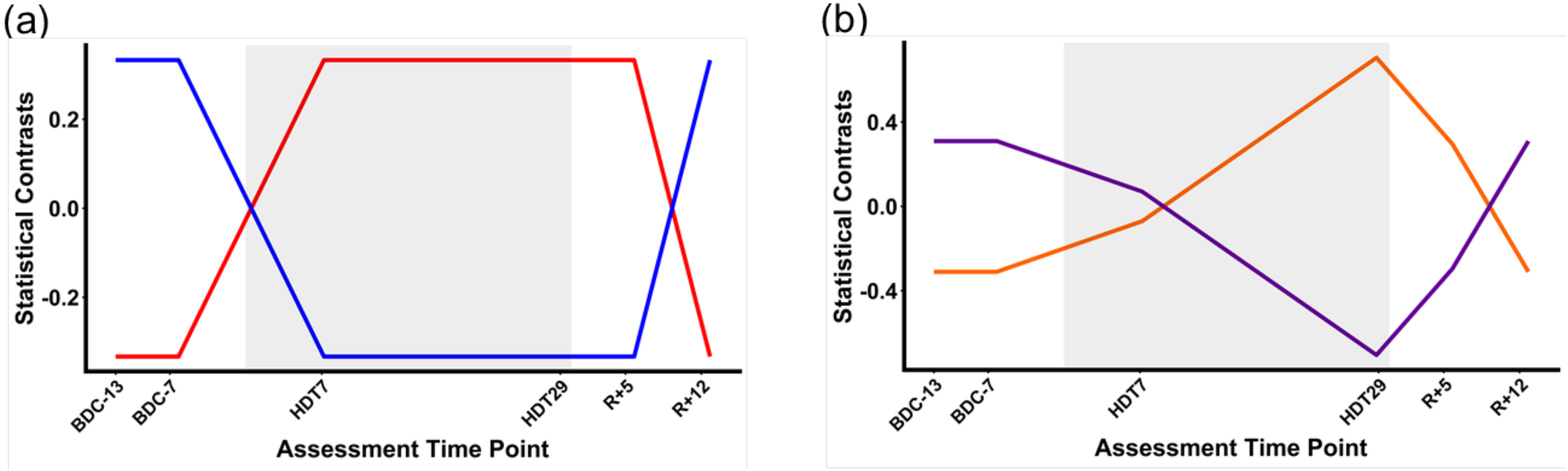

Supplemental Figure 2. Incomplete recovery of (a) Gray matter and (b) FW volumes on R+12. Brain areas where the gray matter and FW volume were significantly increased on R+12 in comparison to BDC-7 are shown in red. Brain areas where the gray matter and FW volume were significantly decreased on R+12 in comparison to BDC-7 are shown in blue.

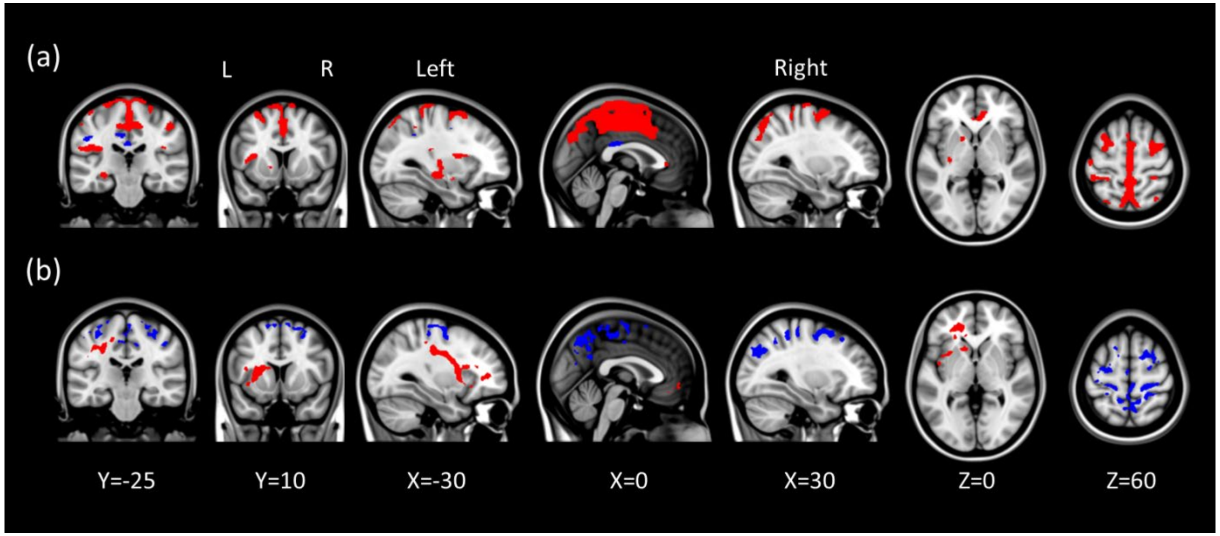

Supplementary Figure 3. Differential effects of the HDBR+CO_2 and HDBR interventions on the (a) 3rd ventricle and (b) cerebellum Crus I volume. (a) The 3rd ventricle volume at each assessment was expressed as a percent of the first baseline measure in order to account for individual differences in head size. (b) The Crus I volume at each assessment was expressed as a percent of the total cerebellum volume obtained during the first baseline measurement in order to account for individual differences in head size. The bed rest phase of the HDBR and HDBR+CO_2 groups are demarcated by shaded boxes of the corresponding color of the groups. The error bars indicate SEM.

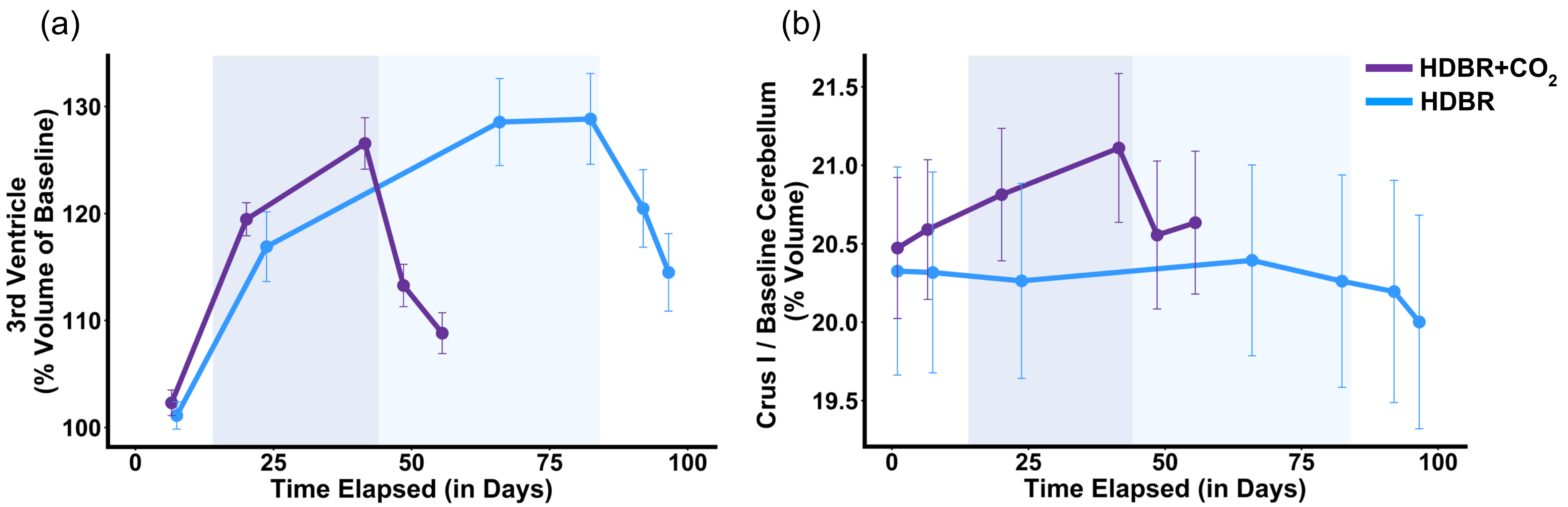

Supplementary Figure 4. Differential effect of HDBR+CO_2 on lateral ventrcial asymmetry between the SANS and NoSANS groups. In order to aid in interpretation the (a) left and (b) right ventricle volume change per SANS subgroup are shown separately. The lateral ventricle volumes are separately normalized to the measure obtained on BDC-13, thus depicting the percent deviation from baseline. The bed rest phase is demarcated in gray. The error bars indicate SEM.

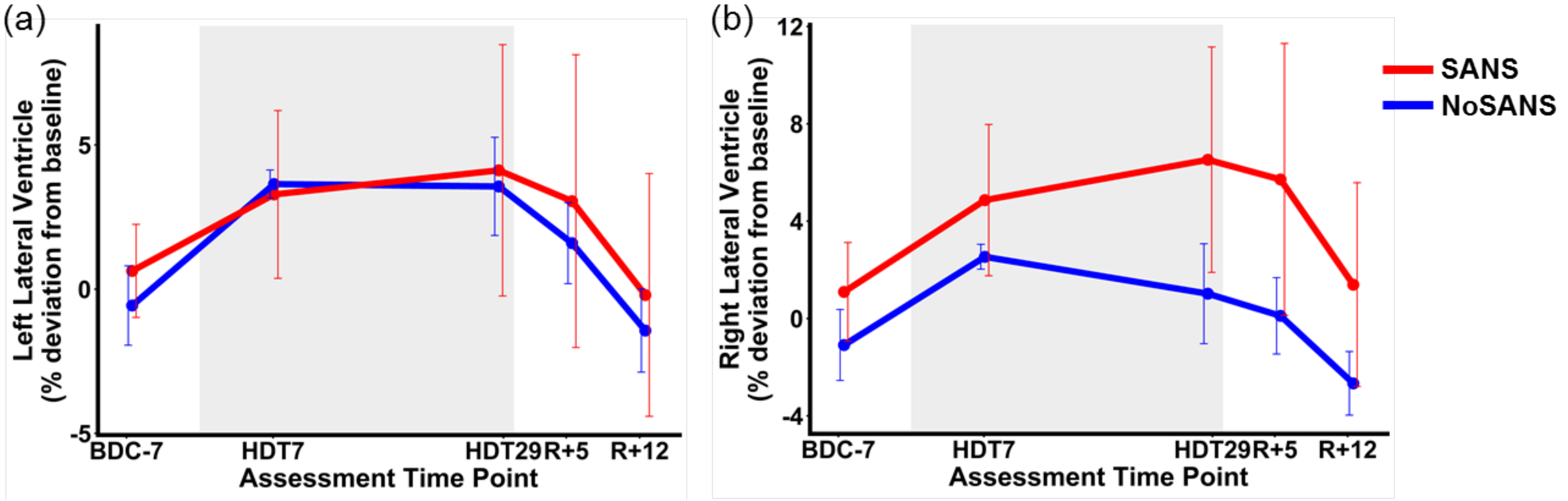

Supplement: Supplementary_Data [file vaperbrain_supplementarymaterials_fin.pdf]
